# The Dynamic Influence of Olorofim (F901318) on the Cell Morphology and Organization of Living Cells of *Aspergillus fumigatus*

**DOI:** 10.3390/jof6020047

**Published:** 2020-04-10

**Authors:** Saskia du Pré, Mike Birch, Derek Law, Nicola Beckmann, Graham E. M. Sibley, Michael J. Bromley, Nick D. Read, Jason D. Oliver

**Affiliations:** 1 F2G Ltd., Lankro Way, Manchester M30 0LX, UK; 2 Manchester Fungal Infection Group, Institute of infection, Immunity and Respiratory Medicine, University of Manchester, CTF Building, Grafton Street, Manchester M13 9NT, UK

**Keywords:** antifungal drugs, olorofim, DHODH, pyrimidines, *Aspergillus fumigatus*, fungal cell morphogenesis

## Abstract

The first characterized antifungal in the orotomide class is olorofim. It targets the de novo pyrimidine biosynthesis pathway by inhibiting dihydroorotate dehydrogenase (DHODH). The pyrimidines uracil, thymine and cytosine are the building blocks of DNA and RNA; thus, inhibition of their synthesis is likely to have multiple effects, including affecting cell cycle regulation and protein synthesis. Additionally, uridine-5′-triphosphate (UTP) is required for the formation of uridine-diphosphate glucose (UDP-glucose), which is an important precursor for several cell wall components. In this study, the dynamic effects of olorofim treatment on the morphology and organization of *Aspergillus fumigatus* hyphae were analyzed microscopically using confocal live-cell imaging. Treatment with olorofim led to increased chitin content in the cell wall, increased septation, enlargement of vacuoles and inhibition of mitosis. Furthermore, vesicle-like structures, which could not be stained or visualized with a range of membrane- or vacuole-selective dyes, were found in treated hyphae. A colocalization study of DHODH and MitoTracker Red FM confirmed for the first time that *A. fumigatus* DHODH is localized in the mitochondria. Overall, olorofim treatment was found to significantly influence the dynamic structure and organization of *A. fumigatus* hyphae.

## 1. Introduction

Invasive fungal infections are threatening the lives of millions of people each year [1]. Although progress has been made, infections of these kind remain difficult to diagnose and treat, and mortality remains unacceptably high [2]. Only four classes of antifungal drugs are currently available on the market [3]. Olorofim (formerly known as F901318) is a novel antifungal belonging to a new class of drugs, called the orotomides, that is currently in phase two of clinical development [4,5,6]. Olorofim selectively inhibits the *de novo* pyrimidine biosynthesis of filamentous fungi by targeting dihydroorotate dehydrogenase (DHODH) [7].

**Figure 1:**
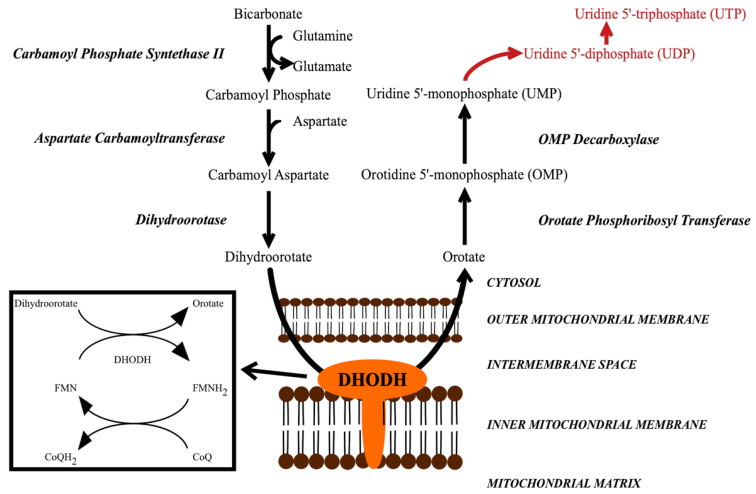
The *de novo* pyrimidine biosynthesis pathway, which leads to the formation of UMP. In subsequent steps, UMP is further converted into UDP and UTP (red words). DHODH catalyzes a redox reaction in which dihydroorotate is oxidized to orotate, while FMN is reduced to FMNH2, which is then re-oxidized by CoQ (ubiquinone). DHODH = dihydroorotate dehydrogenase.

DHODH catalyzes the fourth step in the pyrimidine biosynthesis pathway, the oxidation of dihydroorotate to orotate (Figure 1) [8,9,10,11]. Inhibiting DHODH and pyrimidine biosynthesis leads to the inhibition of the formation of uridine-5′-monophosphate (UMP) and uridine-5′-triphosphate (UTP), which are important for several processes [9,10,12]. They are required for the formation of the DNA/RNA building blocks uracil, cytosine and thymine. UTP is also required for the formation of UDP-glucose and UDP-N-acetyl-glucosamine, the substrates of β-1,3-glucan synthase and chitin synthase, respectively. The products of these reactions, β-1,3-glucan and chitin, form the main components in the structure of the fungal cell wall [13]. UDP-sugars are also required for the formation of glycolipids. Other pyrimidine-charged precursors, such as CDP-diacylglycerol, are important in phospholipid synthesis. Thus, limiting these precursors may influence cellular membrane composition and certain signaling pathways.

Uracil, cytosine and thymine are required to maintain the cell cycle (also known as the duplication cycle in the typically multinucleate hyphae of filamentous fungi because cells do not separate at the end of the mitotic cycle [14]). In the G1 phase, new cellular content is synthesized that is required to accommodate a new round of mitosis. A requirement of this is a significant increase in the availability of pyrimidines [15]. Subsequently, the cell size starts to increase and when a threshold cell volume is reached and sufficient new cell material is present, the cell commits to a new round of mitosis, as has been reported to take place synchronously approximately every 45 min in *A. fumigatus* [16]. After mitosis, new septa are usually formed depending on the size of the hyphal compartments.

A previous study of the effect of olorofim on *A. fumigatus* found a time-dependent decrease in viability, with hyphal swelling leading to cell lysis [17]. The swelling appeared to be accompanied by vacuolar swelling, and this was investigated further in this study. Vacuoles are important contributors to cell size and vacuole dynamics are believed to play a role in cell cycle regulation [18]. They also have multiple other functions in filamentous fungi, including being involved in many aspects of cellular homeostasis, membrane trafficking, signaling, nutrition, long-distance transport, storage of polyphosphates and amino acids, removal of toxic compounds from the cytoplasm, and autophagy [14,19].

The aim of this study was to gain a better understanding of the dynamic intracellular morphological changes that take place in *A. fumigatus* upon exposure to olorofim, in relation to the structure, organization and function of cell walls, mitochondria, vacuoles, nuclei and other organelles labeled with a range of dyes and GFP and imaged by confocal live-cell imaging [20]. The results showed that olorofim treatment leads to several significant morphological rearrangements in *A. fumigatus* hyphae, which interfere with normal cell functioning and over time leads to cell lysis.

## 2. Materials and Methods

### 2.1. Strains

*A. fumigatus* clinical isolate AF293 was used to study the cell wall, septation and vesicles following labeling with a range of live-cell dyes (Table 1). *A. fumigatus* ∆Ku80 (Fungal Genetics Stock Center, A1151) was employed to create a DHODH knock-out strain (ΔpyrE). An *A. fumigatus* cytosolic-GFP expressing strain was also utilized to visualize vacuoles and vesicles by negative staining and for measuring the vacuole/cytoplasm ratio. An *A. fumigatus* strain in which histone 1 was labelled with GFP (H1-GFP) was used to visualize nuclei and mitosis [21]. In order to study the localization of DHODH, a GFP-labelled DHODH strain was constructed under the control of a constitutive gpdA promoter (PgpdA-DHODH-GFP). An overview of the strains used in this study is given in Table S1.

### 2.2. Culture Conditions

All strains were maintained on Sabouraud agar medium at 37 °C. The DHODH deletion strain was maintained on Sabouraud agar medium supplemented with 10 mM uridine and 10 mM uracil. To produce hyphae at an appropriate stage for microscopic analysis, liquid Vogel’s Minimal Medium (VMM) was inoculated with 10^3^ conidia/mL and incubated for 16 h at 37 °C in Ibidi uncoated eight-well chambers (Thistle Scientific, Glasgow, UK). Where indicated, olorofim was dissolved in dimethylsulfoxide (DMSO) at a stock concentration of 0.5 mg/mL and was used at a final inhibitory concentration of 0.1 µg/mL.

### 2.3. Construction of the PgpdA-DHODH-GFP Strain

The DHODH gene from *A. fumigatus* ∆Ku80 was first deleted and replaced by a hygromycin resistance cassette as follows. The *A. fumigatus* DHODH gene (*pyrE*) plus 2050 bp upstream and 2030 bp downstream flanking regions was prepared by PCR using Af293 genomic DNA as a template and JOD5′F1 and JOD3′R1 primers (see Table S2 for primer sequences) with KOD polymerase (Merck Millipore, Watford, UK). The PCR product was purified by Qiaquick purification (Qiagen, Hilden, Germany), A-tailed using Reddy Mix Taq PCR (Sigma-Aldrich, Dorset, UK) and purified again. The 5733 bp product was ligated into pGEMTEasy (Promega, Southampton, UK) to create plasmid PGT_DHODH.

The 5′ and 3′ DHODH flanking regions were amplified by KOD polymerase using PGT_DHODH as a template and primers JOD5′F1 and JODHfus1R giving product DPCR1 and primers JODHfus2F and JOD3′R1 giving product DPCR4. A hygromycin resistance cassette, consisting of the gpdA promoter, hygromycin phosphotransferase and the trpC terminator, was amplified from the pAN7.1 [22] plasmid in two parts—DPCR2 and DPCR3. The primers for DPCR2 were JODHfus1F and HYGr1. The primers for DPCR3 were HYGF1 and JODHfus2R. 

DPCR1 and DPCR2 were joined by fusion PCR [23] using KOD polymerase and primers JOD5′F5 and HYGR2 to yield product DPCR5. DPCR3 and DPCR4 were joined by fusion PCR using primers HYGF2 and JOD3′R5 to yield product DPCR6. DPCR5 and DPCR6 were then joined by fusion PCR using primers JOD5′F4 and JOD3′R4 giving a final product—DKO1.

The knock-out construct DKO1 was transformed into the *A. fumigatus* ∆Ku80 strain by PEG-mediated transformation of protoplasts and inoculated onto SAB plus 1.2 M sorbitol, 10 mM uridine, 10 mM uracil and 200 µg/mL hygromycin followed by incubation at 37 °C for seven days. Colonies obtained were screened by PCR and Southern blotting to confirm that the DHODH gene had been knocked out.

From its sequence it appears that *A. fumigatus* DHODH has an N-terminal mitochondrial targeting sequence. To avoid interference with this targeting sequence, DHODH was C-terminally tagged with GFP. A PgpdA-DHODH-GFP construct was created by fusion PCR as follows (see Table S3 for primer sequences). DHODH plus 1500 bp upstream flanking region was amplified by PCR using primers SP1 and SP2, GFP was amplified from plasmid pFNO3 with primers SP3 and SP4, 1500 bp downstream DHODH flanking region was amplified with primers SP5 and SP6. Using nested primers SP7 and SP8, a DHODH-GFP construct was created by fusion PCR of the SP1-SP2, SP3-SP4 and SP5-SP6 amplicons.

A second fusion PCR was set up to generate a DHODH-GFP construct where the native DHODH promoter was replaced with the constitutive gpdA promoter. The upstream DHODH flanking region without the native DHODH promoter was amplified with primers SP9 and SP10, the gpdA promoter was amplified from plasmid pAN7.1 with primers SP11 and SP22, DHODH-GFP was amplified from the first fusion PCR product with primers SP23 and SP8. Using nested primers SP7 and JOD3′R3, a gpdA-DHODH-GFP construct was created by fusion PCR of the SP9-SP10, SP11-SP22 and SP23-SP8 amplicons. 

Phusion Hot Start II High Fidelity Polymerase (Thermo Scientific) was used for PCR. Fusion constructs were extracted from an agarose gel with Qiaquick Gel Extraction Kit (Qiagen), A-tail added with DreamTaq Green DNA polymerase and the resulting construct ligated into a pGEM-T easy vector (Promega). The gpdA-DHODH-GFP construct was transformed into the DHODH knock-out strain, replacing the hygromycin resistance cassette, by PEG-mediated transformation of protoplasts and inoculated onto SAB plus 1.2 M sorbitol followed by incubation at 37 °C for 5–7 days. Colonies obtained were screened by PCR to confirm integration of the gdpA-DHODH-GFP construct.

For a graphic overview of the constructed strains, please see Figure S1.

### 2.4. Probes for Live-Cell Imaging

The live-cell probes, their concentrations, selectivities, sources, and wavelengths for excitation and emission detection are shown in Table 1. All dyes were dissolved in DMSO, except for Aniline Blue (50 mM phosphate buffer pH 8, [24]) and Calcofluor White (water). Dyes were added to the *A. fumigatus* cultures for 30–60 min at 37 °C prior to imaging.

### 2.5. Live-Cell Confocal Microscopy

All images were captured with a Leica SP8X confocal microscope, using the Leica LAS AF software for image acquisition. They were acquired using either a 40×/0.85 NA dry objective or a 63×/1.2 NA water objective. The wavelengths used to excite and detect the fluorescence, and the lasers used are shown in Table 1. The FIJI distribution package of ImageJ (v 1.52c)was utilized for image processing and measurement [25].

### 2.6. Statistics

Statistical analyses were performed in GraphPad Prism 7. Student’s *t*-tests were performed with 99% confidence intervals. All experiments were done in triplicate. * indicates a significant difference to the control (*p* < 0.01). Bars represent the standard deviations (SD).

## 3. Results

### 3.1. A. fumigatus Has a Class 2 DHODH Located in the Mitochondria

**Figure 2:**
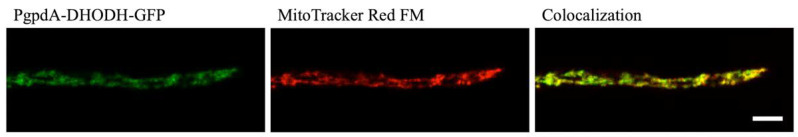
Colocalization of PgpdA-DHODH-GFP with the mitochondrion-selective dye MitoTracker Red FM indicates that *A. fumigatus* has a class 2 DHODH that is localized in the mitochondria. Scale bar = 5 µm.

DHODH enzymes are classified as belonging to class 1 or class 2 depending on structure, cellular location and the nature of the terminal redox cofactor [11]. Although most eukaryotes have a class 2 DHODH located at their mitochondria, some fungi, including *Saccharomyces cerevisiae*, carry a cytosolic class 1 DHODH. To predict the class of DHODH present in *A. fumigatus*, bioinformatics tools Predotar [26], TargetP [27] and MitoFates [28] were used to predict whether an N-terminal mitochondrial targeting sequence (MTS) is present in the protein sequence of DHODH (Afu2g11010). All three of these applications predicted that *A. fumigatus* DHODH possesses an MTS, suggesting it is a class 2 DHODH. To confirm that DHODH is localized in the mitochondria, a colocalization study was carried out in which MitoTracker Red FM was used to stain mitochondria in an *A. fumigatus* strain in which DHODH was labelled with GFP under the expression of a constitutive gpdA promoter. The MitoTracker Red FM and the DHODH-GFP signal showed colocalization patterns consistent with the GFP-tagged DHODH being present in the mitochondria (Figure 2). This confirms that *A. fumigatus* has a class 2 DHODH associated with its mitochondria.

### 3.2. Olorofim Treatment Leads to Cell Wall Remodeling

As pyrimidines are involved in the formation of the cell wall components β-1,3-glucan and chitin (a polymer of β-1,4-N-acetyl-d-glucosamine), the cell wall content of these two components was investigated. Aniline Blue was used to stain β-1,3-glucan and chitin was stained with Calcofluor White (CFW) (Table 1). Hyphae were obtained by pre-incubating conidia for 16 h in VMM, following which they were treated with 0.1 µg/mL olorofim. After 24 h, treated and untreated hyphae were stained with Aniline Blue or CFW and the relative fluorescence intensity of the dyes was measured as an estimate of the amount of β-1,3-glucan and chitin in the cell wall. 

**Figure 3:**
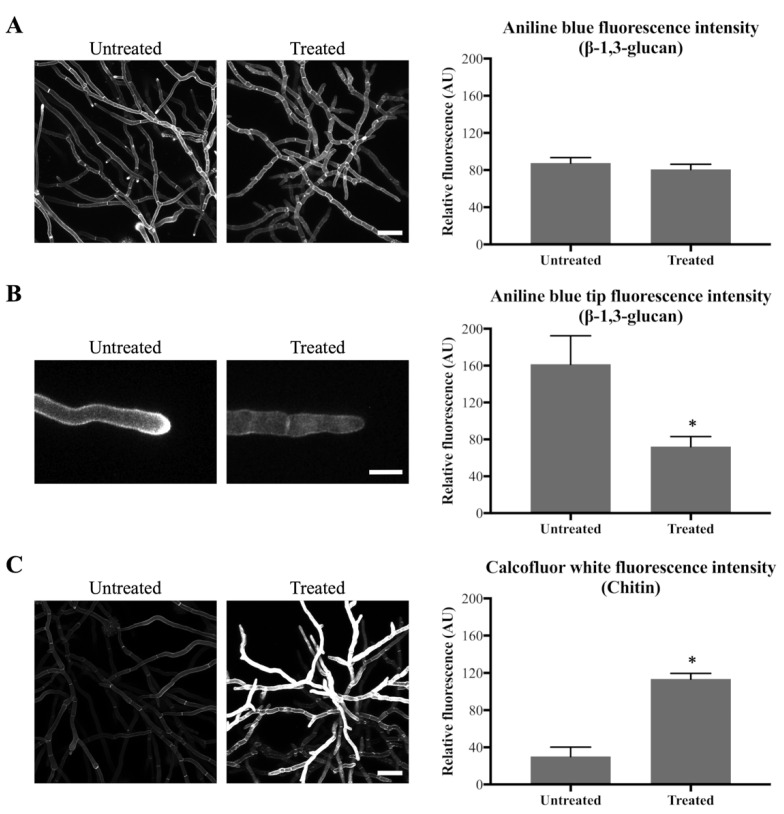
Olorofim causes cell wall remodeling as indicated by Aniline Blue (β-1,3-glucan) and CFW (chitin) staining. Fluorescence images and measurements are shown on the left and right panels, respectively; error bars represent the SD: (**A**) Aniline Blue (β-1,3-glucan) staining. No difference in overall fluorescence intensity of mycelial hyphae before and after treatment with 0.1 µg/mL olorofim for 24 h. Scale bar = 40 µm; (**B**) Aniline Blue (β-1,3-glucan) staining. Reduction in β-1,3-glucan in hyphal tips at the colony periphery following treatment. Scale bar = 10 µm; (**C**) CFW (chitin) staining. Chitin content was increased in the treated hyphae throughout the mycelium. Scale bar = 40 µm. * *p* < 0.01.

After 24 h of olorofim exposure, no significant decrease in overall β-1,3-glucan content within the mycelium as a whole was observed in the cell walls (Figure 3A). However, a significant decrease was observed specifically at the hyphal tips at the periphery of the treated mycelium, with a 2.2-fold decrease in Aniline Blue fluorescence (Figure 3B). Chitin content within the mycelium was significantly increased, with a 3.8-fold increase in CFW fluorescence after 24 h treatment with olorofim (Figure 3C). This result demonstrates that olorofim treatment leads to cell wall remodeling by changing the β-1,3-glucan in hyphal tips and chitin content throughout the mycelium.

### 3.3. Olorofim Exposure Increases Hyphal Septation

As previously reported, additional septa were formed in olorofim treated germ tubes and hyphae [17]. To investigate this further, the interseptal distance was measured in hyphae that were stained with Aniline Blue and CFW.

**Figure 4:**
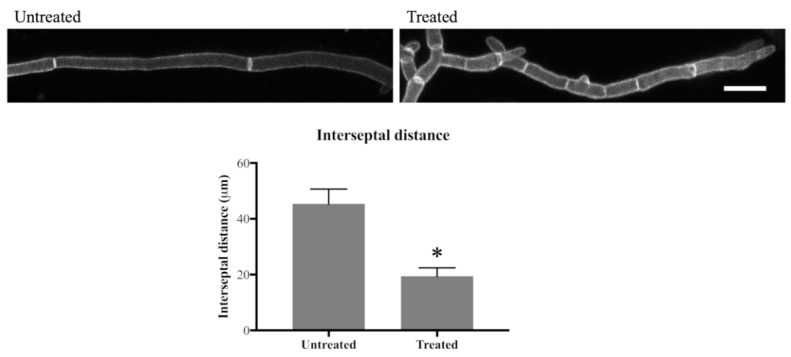
Olorofim treatment decreases interseptal distance indicating that septum formation is increased. Scale bar = 20 µm. Error bars represent SD. * *p* < 0.01.

In the untreated hyphae, septa were, on average, 45 µm apart, whereas in 24 h-treated hyphae this distance was 19 µm, which translates to 2.2 septa/100 µm in untreated hyphae and 5.3 septa/100 µm in olorofim-treated hyphae, an increase of >2-fold in the number of septa after drug treatment (Figure 4). This result indicates that olorofim treatment results in increased septation coupled with a reduction in the size (shortening) of hyphal compartments.

### 3.4. Vacuolar Volume Increases upon Olorofim Exposure

Exposure to olorofim caused hyphae to significantly increase in size by swelling [17]. Enlarged vacuoles were observed in olorofim-treated conidia and hyphae. To further investigate this, vacuoles were stained with Cell Tracker Blue (CMAC) in a cytoplasmic GFP expressing strain of *A. fumigatus*. The volumes of the cytoplasm and the vacuoles in subapical compartments were measured and vacuole/cytoplasm ratios determined.

**Figure 5:**
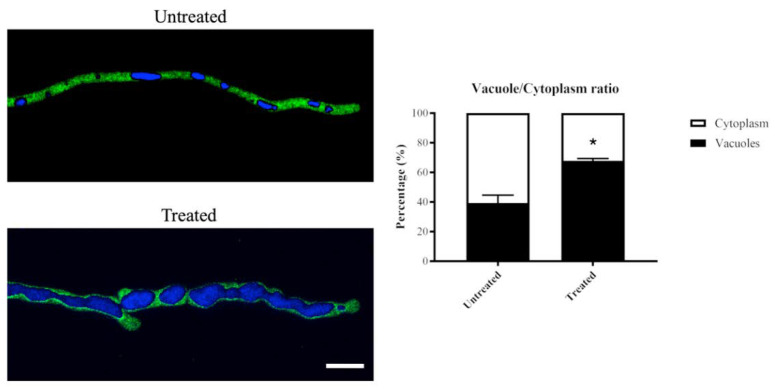
Vacuolar volume increases in 24 h olorofim-treated hyphae. Vacuoles in a cytoplasmic-GFP strain were stained with CMAC and the vacuole/cytoplasm ratio was measured. Vacuolar volume was significantly enlarged after exposure to olorofim, resulting in a reduced cytoplasmic volume. Scale bar = 10 µm. Error bars represent SD. * *p* < 0.01.

In the hyphae exposed to olorofim for 24 h, vacuoles underwent a significantly greater increase in volume than the cytoplasm (Figure 5). In the untreated hyphae, vacuoles took up, on average, 39% of the cytoplasm; in the 24 h-treated hyphae, this was nearly 68%. This is a 1.7-fold increase in vacuolar volume in the treated hyphae.

The vacuoles in the treated hyphae appeared to be fused together and form large globose organelles, whereas in untreated hyphae, various shapes (tubular and spherical/ovoid) and sizes of vacuoles were observed (Figure 5). 

These data demonstrated that olorofim treatment leads to the formation of large vacuoles that take up a significant proportion of the hyphal compartments, decreasing the volume of the cytoplasm.

### 3.5. Prolonged Olorofim Exposure Leads to Inhibition of Mitosis

Pyrimidine biosynthesis is important for DNA/RNA synthesis and cell cycle regulation. Therefore, the effect of olorofim on nuclei was studied using an *A. fumigatus* strain in which the nuclei were tagged with GFP (H1-GFP strain). Nuclei were observed over a course of 2 h to observe the dynamic effects on nuclear morphology and mitosis after adding olorofim.

**Figure 6:**
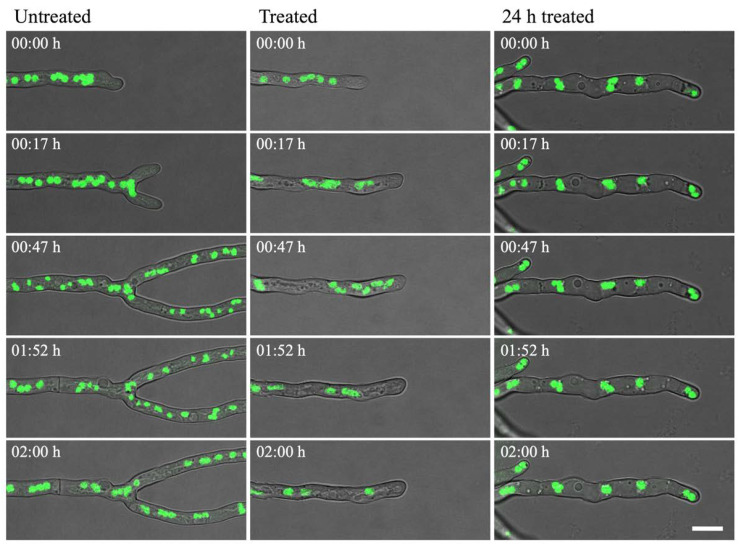
H1-GFP hyphae were incubated either in the absence (untreated) or presence (treated; 24 h treated) of 0.1 µg/mL olorofim. The nuclei (green) were observed from 0–2 h (untreated; treated) or from 24–26 h (24 h treated). In untreated hyphae, nuclei moved at a steady rate, which increased 15–20 min after addition of olorofim. In hyphae treated for 24 h, nuclear motility was almost static. Scale bar = 10 µm.

In the untreated control, nuclei were regularly shaped during interphase and decreased in size during nuclear division, after which normal size for interphase was regained (Figure 6 left column; Video S1). Nuclei moved within the cytoplasm at a steady rate, which was slightly increased just after mitosis. Two rounds of mitosis were observed in Video S1. When olorofim was added, a clear effect was seen on nuclear motility from 15–20 min after addition when the nuclei started to display rapid movements and mitosis could not be observed. However, ‘normal’ motility was restored 1–2 h after the drug was added (Figure 6 middle column; Video S2). In hyphae that had been treated with olorofim for 24 h, nuclei were almost static during the 2h that followed. Hyphal growth had been completely halted by olorofim at this stage and the number of nuclei in this section of hyphae does not change in the 2 h observation period, indicating that mitosis has been inhibited (Figure 6 right column; Video S3).

**Figure 7:**
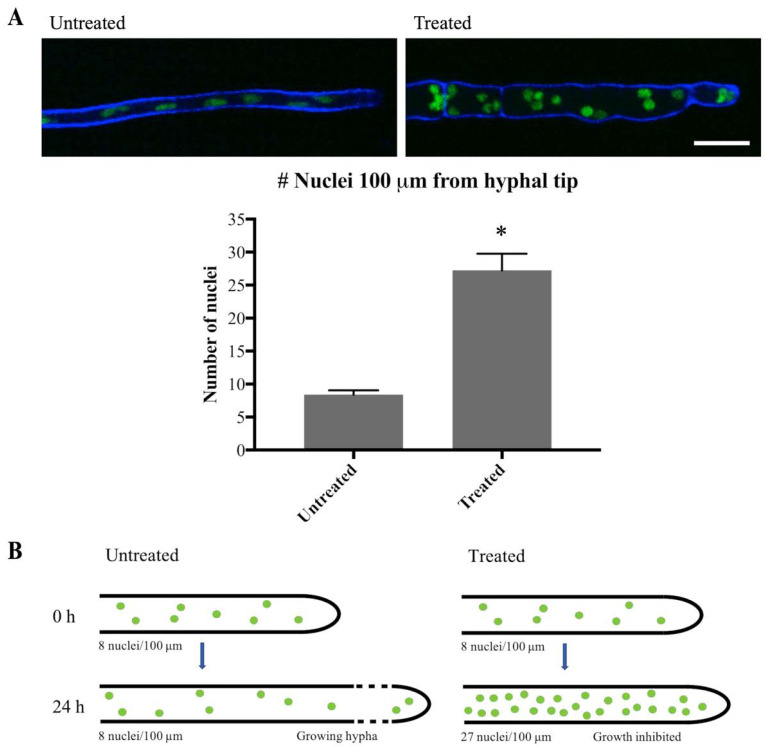
Olorofim treatment inhibits mitosis. (**A**) Imaging of H1-GFP nuclei within 100 µm of the hyphal tip at 0 h (Untreated) and 24 h (Treated) following addition of olorofim. Scale bar = 10 µm. Error bars represent SD. (**B**) After 24 h of olorofim treatment, only 1–2 rounds of mitosis are estimated to take place, based on the accumulated number of nuclei in the hyphal tip. In untreated hyphae, newly formed nuclei move along with the growing hyphal tip, resulting in more equally dispersed nuclei. * *p* < 0.01.

To confirm that mitosis was indeed inhibited, the number of nuclei close to the hyphal tip was quantified at 0 h and 24 h after addition of olorofim. Generally, in filamentous fungi nuclei are equally dispersed in the hyphae and move along with the growing hyphal tip, as observed in Video S1. As previously described [17], hyphal elongation is inhibited by olorofim (Figure 6 and compare Video S2 versus S1), so the hyphae exposed to olorofim extend very little in 24 h compared to untreated hyphae. For this reason, it was hypothesized that the nuclei present at 0 h and those additionally formed by mitosis over 24 h of treatment, cannot move along a growing hyphal tip and will therefore accumulate (Figure 7B). Hence, the number of nuclei observed at the hyphal tip at 24 h can be compared to the number of nuclei at the hyphal tip at 0 h to estimate the number of rounds of mitosis that had occurred. At 0 h, over a distance of 100 µm from the hyphal tip an average of eight nuclei were counted. At 24 h, an average of 27 nuclei were counted (Figure 7A) in the hyphal tip indicating that either one or two rounds had taken place during 24 h of olorofim treatment. In 2 h-time course movies when no olorofim was added, such as Video S1, mitosis occurred approximately every 60–75 min, which equates to at least 19 rounds of mitosis over a 24 h period. Thus olorofim-treated hyphae showed a marked reduction in mitotic rounds from 19 to 1–2 over a 24-h period (Figure 7B). This indicates that olorofim inhibits mitosis, consistent with cell cycle arrest.

### 3.6. Olorofim-Treated Hyphae Contain Unidentified, Vesicle-Like Structures

**Figure 8:**
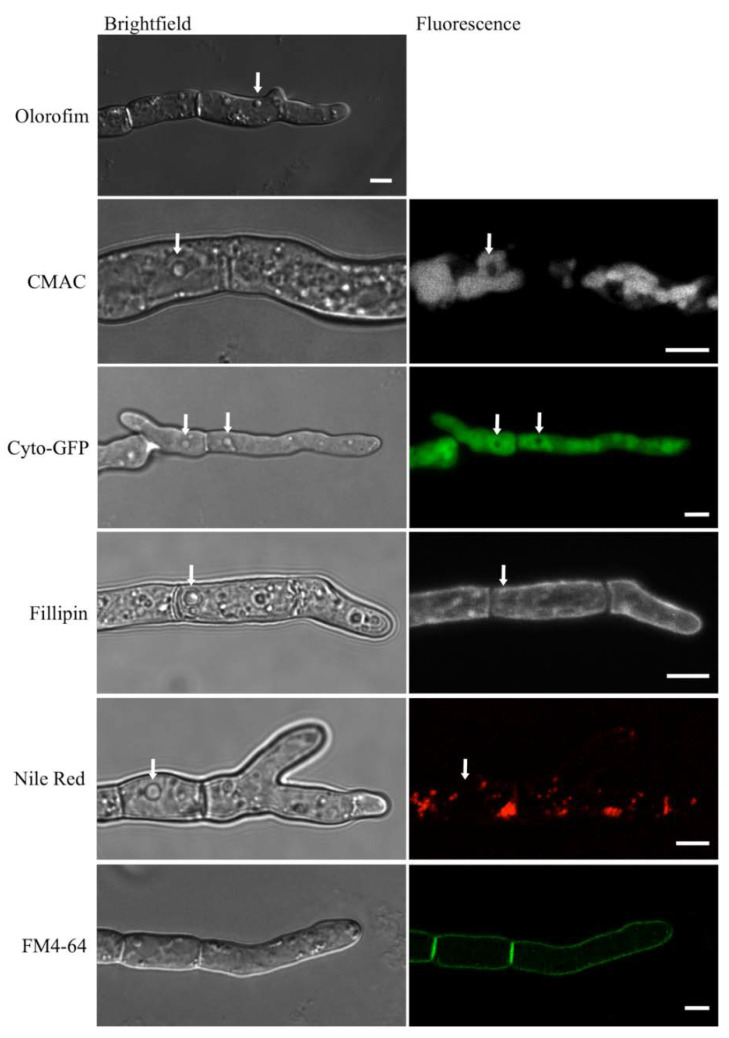
Unidentified, vesicle-like structures were detected in olorofim-treated hyphae. They are readily identified in unstained DIC images (arrow) but are not stained with CMAC, Filipin, negative staining with cytosolic GFP (cyto-GFP), Nile Red (arrows) or FM4-64. Scale bar = 5 µm.

In hyphae that had been treated with olorofim, distinctive vesicle-like structures could be observed, which were absent from untreated hyphae (indicated with arrows in Figure 8, top image). When hyphae were stained with the vacuole-selective stain, CMAC (Table 1), the vesicle-like structures remained unstained and thus appeared to be distinct from the vacuoles (Figure 8, second row). Likewise, when a cytosolic-GFP (Cyto-GFP) strain of *A. fumigatus* was treated with olorofim, no fluorescence was observed in the vesicles (they were negatively stained), indicating that the vesicles were physically separate from the cytosol (Figure 8, third row; Table 1). The vesicular structures were not stained by Filipin, indicating that their membranes lacked sterol (Figure 8, fourth row; Table 1). Neither did they colocalize with Nile Red, excluding the possibility of the structures being lipid droplets or containing neutral lipids (Figure 8, fifth row; Table 1). FM4-64, a membrane-selective dye, only stained the periphery of the hyphae (Figure 8, sixth row; Table 1). These results show that the observed vesicle-like structures are distinct from both the cytoplasm and the vacuoles, but their exact nature remains to be identified.

## 4. Discussion

In this study, the dynamic effects of the novel antifungal drug olorofim (F901318) on the intracellular structure and organization of the human pathogen *A. fumigatus* were investigated. Although the target of olorofim, DHODH, has been described [7], the downstream morphological consequences for the fungal cell, following exposure to the drug, are unknown. Therefore, the effects of olorofim treatment on subcellular organelle organization and function was investigated by using fluorescent staining or tagging with GFP to be able to microscopically investigate their morphological changes upon drug treatment. The structures were investigated either directly after addition of the drug (nuclei) or after 24 h (all investigated organelles). The 24 h timepoint was chosen as it was previously shown that hyphal lysis, staining with the viability dye DiBac and observation of vacuolar swelling were apparent after 24 h exposure to olorofim [17].

There are two classes of DHODH recognized, class 1 and class 2, based on structure, subcellular location and redox cofactor used. It was presumed that the *A. fumigatus* DHODH was a class 2 type located in the mitochondria and this was supported by bioinformatics tools that predicted a high probability of an MTS being present in the sequence. Colocalization of a GFP-tagged DHODH and the mitochondrial dye MitoTracker Red FM confirmed that *A. fumigatus* DHODH is indeed localized in the mitochondria, confirming the initial prediction of a class 2 DHODH. 

Pyrimidines are known to be important for the construction of the cell wall via intermediary UDP-sugars [13]. In order to investigate the effects of UDP-sugar depletion on the cell wall structure following exposure to olorofim, β-1,3-glucan and chitin were stained with Aniline Blue and CFW, respectively, and the fluorescence intensity was measured. No significant difference was found in overall Aniline Blue fluorescence, but specifically at the hyphal tip, the site of active growth, a significant decrease was observed. In actively growing hyphae, the addition of new cell wall material is constantly needed. Olorofim inhibits pyrimidine biosynthesis, which presumably leads to a reduction in the availability of UTP. UTP is required for the synthesis of UDP-glucose, the substrate of β-1,3-glucan synthase. A decrease in β-1,3-glucan synthesis probably resulted in the observed absence of Aniline Blue accumulation in the tips of the treated hyphae and likely caused an inhibition of hyphal growth [17].

In contrast to β-1,3-glucan, chitin content was significantly increased upon exposure to olorofim. Potentially, this chitin upregulation is a rescue mechanism of the fungus to avoid cell lysis. It does appear to be contradictory as UTP is required for the formation of chitin. Chitin upregulation is possibly a relatively quick stress response after the addition of olorofim when sufficient pyrimidines are still available or perhaps it is regulated by an alternative cell wall salvage mechanism. 

Interestingly, increased chitin content was also previously reported in *A. fumigatus* treated with caspofungin [29], an echinocandin which targets β-1,3-glucan synthase. Other similarities with caspofungin-treated hyphae include hyphal swelling and cell lysis (see [30]), although in the case of caspofungin, the cell lysis was hyphal tip specific, whereas olorofim-induced lysis can occur at any part of the cell [17]. Additionally, increased septation, observed in caspofungin-treated hyphae, was also observed in olorofim-treated hyphae, which again could be a potential stress response.

The effects of olorofim (and some of caspofungin’s effects) on the cell wall are in contrast to those recently observed following voriconazole treatment of *A. fumigatus* [31]. Geiβel et al. observed the formation of carbohydrate patches containing β-1,3-glucan and chitin at specific sites in the hyphae. These patches colocalise with (and potentially cause) invaginations in the cell membrane that precede cell lysis events. No such patches or membrane invaginations are seen following olorofim-treatment, with hyphae swelling prior to lysis [17] indicating a potential build-up of pressure pushing out rather than inward pressure in. It appears that olorofim’s effects on *A. fumigatus* have more in common with voriconazole’s effects on the morphology of *Lomentaspora prolificans* [32]. After 9 h of incubation with voriconazole, hyphal length was decreased, while hyphal width was increased compared to controls. These are similar results to those we previously described for olorofim-treated *A. fumigatus* [17]. Furthermore, in the same study they also describe increased CFW fluorescence in voriconazole-treated *L. prolificans* cells, as we have found in this study for *A. fumigatus* after incubation with olorofim, further suggesting it may be a common response to antifungal challenge.

Olorofim-treated hyphae were found to contain enlarged vacuoles. In filamentous fungi, vacuolar appearance is highly dynamic and depends on the state of the fungus. Older hyphal compartments usually contain enlarged vacuoles, while younger compartments contain long tubular vacuoles that play a role in multiple functions, including cell signaling and cell biogenesis; hyphal tips usually do not contain any vacuoles. It is believed that under nutrient-limited conditions, large vacuoles are formed to decrease the volume of the cytoplasm, which decreases the need for nutrients and protein synthesis [19]. Furthermore, compartments containing large vacuoles in *Candida albicans* were previously found to be cell cycle arrested. The cytoplasmic volume is believed to be an important trigger for the G1 start event. As large vacuoles decrease the volume of the cytoplasm, highly vacuolated compartments are therefore believed to be arrested in G1. When olorofim was added to an H1-GFP strain, nuclear motility increased for 1–2 h following addition of the drug, before returning to previous levels. In the 24 h after the addition of olorofim, only one or two rounds of mitosis take place and it appears that no further mitosis occurs after 24 h of treatment. Olorofim blocks the formation of the following pyrimidines found in DNA and RNA: cytosine, thymine and uridine. It is reasonable to assume that a decrease in the cellular levels of pyrimidines may lead to an inability to replicate the full set of DNA, which blocks the onset of mitosis and possibly causes cell cycle arrest.

The formation of large vacuoles could also be a sign that autophagy is activated in response to olorofim treatment, especially as vesicle-like structures have been observed in treated hyphae. Autophagy is a form of programmed cell death, in which toxic cytoplasmic contents are sequestered in so called autophagic bodies (autophagosomes), which fuse with the vacuole, within which the contents are released and subsequently degraded [33]. This leads to the formation of larger vacuoles, as observed in olorofim-treated hyphae. The presence of autophagic bodies is an important hallmark for autophagy, which is why the vesicle-like structures in treated hyphae were further investigated. Treated hyphae were stained with fluorescent probes selective for vacuoles (CMAC), sterol (Filipin) and neutral lipids (Nile Red), and additionally, a cytosolic-GFP strain was utilized. It was clear that the vesicles were excluded from both vacuoles and the cytosol. The structures were also not stained by Filipin or Nile Red, and thus it was not possible to determine the nature of these vesicle-like structures. To truly determine whether the vesicles are autophagosomes, and thus if autophagy is activated by olorofim, a strain should be utilized in which the autophagy-related protein 8 (ATG8) is tagged with GFP because ATG8 is associated with autophagosomes [33,34].

Previously, it was demonstrated that prolonged exposure of *A. fumigatus* to olorofim was fungicidal, with hyphal swelling leading to cell lysis [17]. The results from this study show that olorofim treatment significantly influences the dynamic structure and organization of hyphae. It is clear that pyrimidines are crucial for many cellular processes and not just DNA/RNA. Hence, drugs that interfere with the pyrimidine biosynthesis pathway, such as the orotomides, have the potential to be a very useful addition to the tools available for antifungal therapy.

## Figures and Tables

**Table 1:** Live-cell probes, their selectivities, concentrations used, commercial sources and confocal microscope imaging parameters used.

Probes	Selectivity	Concentration	Commercial Source	Excitation Wavelength	Emission Wavelength	Excitation Source
Mitotracker Red FM	Mitochondria	500 ng/mL	Molecular Probes	581 nm	595–650 nm	White light laser
Aniline Blue	Cell wall	25 µg/mL	Sigma-Aldrich	405 nm	420–470 nm	Blue diode laser
Calcofluor White (CFW)	Cell wall chitin	0.1 µg/mL	Sigma-Aldrich	405 nm	420–470 nm	Blue diode laser
Wheat germ agglutinin-orange (WGA-orange)	Cell wall chitin	5 µg/mL	Molecular Probes	555 nm	570–630 nm	White light laser
Cell Tracker Blue/CMAC	Vacuoles	1 µM	Molecular Probes	405 nm	420–470 nm	Blue diode laser
Filipin	Cell membrane ergosterol	1 µg/mL	Sigma-Aldrich	405 nm	420–470 nm	Blue diode laser
Nile Red	Membrane lipids, neutral lipid droplets	10 µg/mL	Sigma-Aldrich	514 nm	530–600 nm	White light laser
FM4-64	Membrane	5 µM	Molecular Probes	515 nm	550–700 nm	White light laser
GFP	Various	Not applicable	This study	488 nm	500–550 nm	White light laser

## Supplementary Materials

The following are available online at https://www.mdpi.com/2309-608X/6/2/47/s1, Figure S1: Overview of the strain construction strategies, Table S1: Strains used in this study, Table S2: Primer sequences used for DHODH knock-out strain, ∆pyrE, Table S3: Primer sequences used for PgpdA-DHODH-GFP strain, Video S1: H1-GFP untreated, Video S2: H1-GFP treated, Video S3: H1-GFP 24 h treated.
