# Supplementary material for: The Dynamic Influence of Olorofim (F901318) on the Cell Morphology and Organization of Living Cells of Aspergillus fumigatus
Source: J Fungi (Basel). 2020 Apr 10;6(2):47. doi: 10.3390/jof6020047 (PMC7345704; doi:10.3390/jof6020047)
Supplement: Supplementary file 1 [file jof-06-00047-s001.zip › Supplementary files/Supplementary data.docx]

**Supplementary data**

**Table S1.** Strains used in this study.

| **Strain** | **Function** | **Genotype** | **Parent strain** | **Source** |
| --- | --- | --- | --- | --- |
| **^AF293^** | ^Clinical isolate^ |  |  | ^NCPF 7367^ |
| ***^AKUB^*^KU80^** | ^Non-homologous recombination mutant^ | ^∆^*^ku80^* | ^CEA17^ | ^(1)^ |
| **^∆pyrE^** | ^DHODH deletion^ | ^∆a^*^kuB::hph::pyrE^*^-^ | *^AKUB^*^KU80^ | ^This study^ |
| **^PgpdA-DHODH-GFP^** | ^DHODH-GFP, constitutive promoter^ | ^∆a^*^kuB::^*^[P^*^gpdA-pyrE-gfp^*^]^ | ^∆pyrE^ | ^This study^ |
| **^GFP-pyrG^** | ^Cytoplasmic GFP^ | ^∆a^*^kuB^*^::^*^ßtub::gfp::pyrG+^* | *^AKUB^*^KU80^ | ^MFIG^ |
| **^H1-GFP^** | ^Nuclear GFP^ | ^∆a^*^kuB::^*^[P^*^gpdA-H1-sgfp^*^]^ | *^AKUB^*^KU80^ | ^MFIG, (2)^ |

**
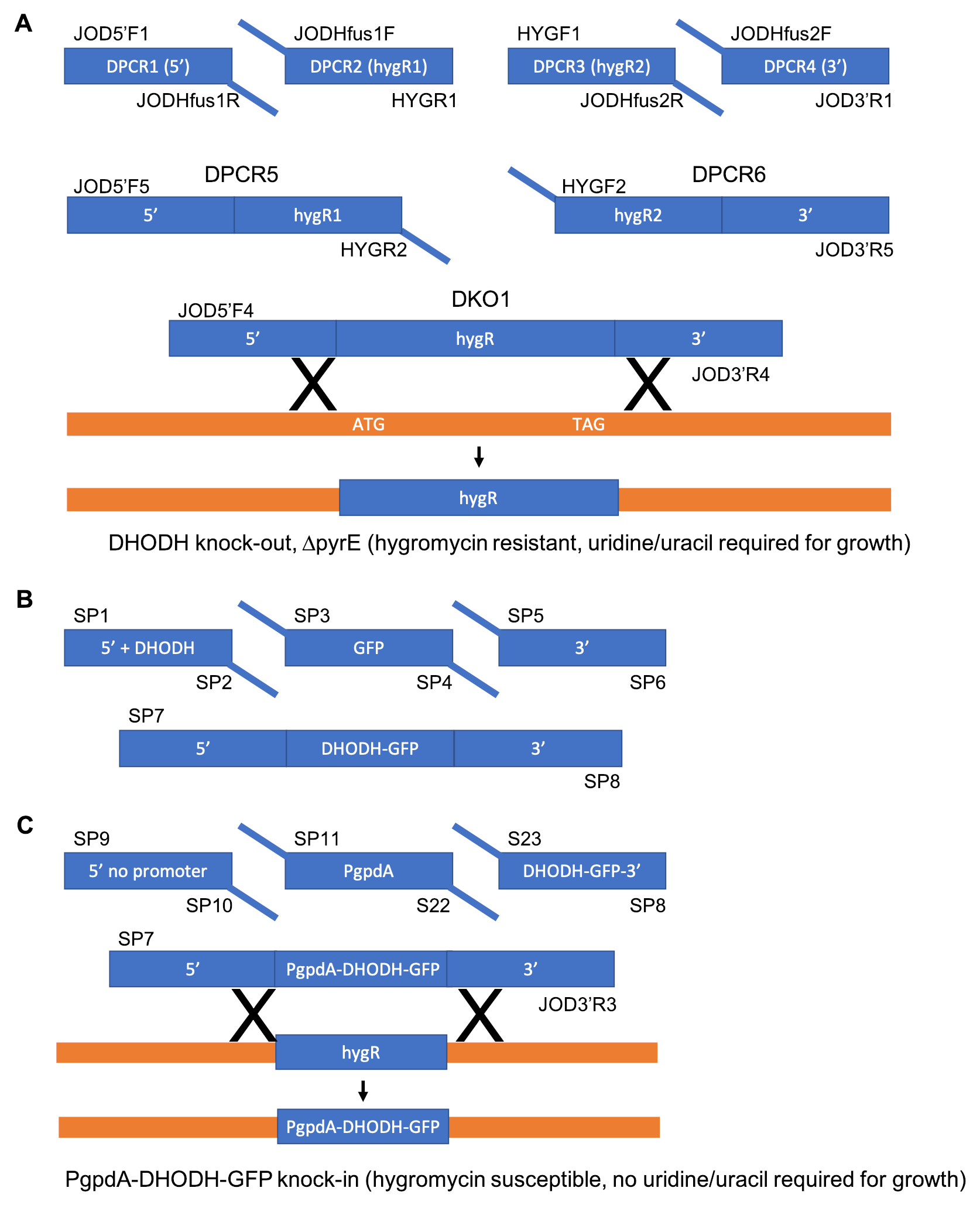
**

**Figure S1.** Overview of the strain construction strategies. (**a**) DHODH knock-out strain (∆pyrE), (**b**) DHODH-GFP fusion PCR and (**c**) PgpdA-DHODH-GFP strain.

**Table S2.** Primer sequences used for DHODH knock-out strain, ∆pyrE.

| **Primer name** | **Primer sequence** |
| --- | --- |
| HYGF1 | CCATCCTTCCCATCCCTTAT |
| HYGR1 | GAGACGCTGTCGAACTTTT |
| HYGF2 | TCAGTTCGAGCTTTCCCACT |
| HYGR2 | AGCGGGGTAGCTGTTAGTCA |
| JOD5’F1 | TCGAAGTTCCACTTGGCTTT |
| JOD3’R1 | TAGATGCCATGCCAGAAACA |
| JOD5’F4 | CTCTGCTCCCCGATCTACAG |
| JOD3’R4 | GGCTGGAGCTGACTCTTGAC |
| JOD5’F5 | GCCCAGAAGCTAATTCGATG |
| JOD3’R5 | TCCCTCTGACATCCGAGTTC |
| JODHfus1R | GTAGAGATACAAGGGAATTCGACGAGTCTGCCTTGTCAGG |
| JODHfus2F | CACTCCACATCTCCACTCGAACTGTTTCGTTTGGATACAG |
| JODHfus1F | CCTGACAAGGCAGACTCGTCGAATTCCCTTGTATCTCTAC |
| JODHfus2R | CTGTATCCAAACGAAACAGTTCGAGTGGAGATGTGGAGTG |

**Table S3.** Primer sequences used for PgpdA-DHODH-GFP strain. Lower case sequence indicates the linkers used for fusion PCR.

| **Primer name** | **Primer sequence** |
| --- | --- |
| SP1 | GCCGACAAAACGAAATCACAG |
| SP2 | ggcaccggctccagcgcctgcaccagctcc  TTGACGGTTTTTCTTTTCCTCTCG |
| SP3 | ggagctggtgcaggcgctggagccggtgccatg  AGTAAAGGAGAAGAACTTTTCAC |
| SP4 | atccacttaacgttactgaaatccta  TTTGTATAGTTCATCCATGCC |
| SP5 | gatttcagtaacgttaagtggat  ACTGTTTCGTTTGGATACAGCG |
| SP6 | CCAAGTGCGTATTGTCCATTCATC |
| SP7 | CTGCTCCCCGATCTACAGG |
| SP8 | CTGGAGCTGACTCTTGACTCC |
| SP9 | ATCCTACTGACGTTGCTGTG |
| SP10 | gggagcatatcgttcagagc  CTATTCGTCACTCATCTTGTCTTCG |
| SP11 | gctctgaacgatatgctccc  GAATTCCCTTGTATCTCTACACAC |
| SP22 | ccggctcggtaacagaacta  GGTTCTTGGATGGGAAGATG |
| SP23 | tagttctgttaccgagccgg  ATGGTTGCCAATTCCACCAG |
| JOD3’R3 | ACTCAAGCCGAACGATCAAT |

**Legends supplementary movies**

**Video S1.** H1-GFP untreated

Using an H1-GFP strain, nuclear morphology was observed over a period of 2 h, without the addition of drugs. Nuclei appeared regularly shaped during interphase and decreased in size during nuclear division, after which normal size for interphase was regained. Nuclei moved within the cytoplasm at a steady rate, which was slightly increased just after mitosis.

**Video S2.** H1-GFP treated

Using an H1-GFP strain, nuclear morphology was observed over a period of 2 h, after the addition of 0.1 μg/ml olorofim. 15-20 min after addition of the drug, nuclear motility was disrupted, with nuclei starting to display rapid movements. ‘Normal’ motility was restored 1-2 h after the drug was added.

**Video S3.** H1-GFP 24 h treated

An H1-GFP strain that was treated for 24 h with 0.1 μg/ml olorofim, was observed over a period of 2 h. Nuclei were almost static and no mitosis was observed.
